# Supplementary figures and images for: Symbiotic bacteria may support calcium carbonate precipitation in the Gulf toadfish
Source: PLoS Biol. 2026 May 18;24(5):e3003764. doi: 10.1371/journal.pbio.3003764 (PMC13215605; doi:10.1371/journal.pbio.3003764)

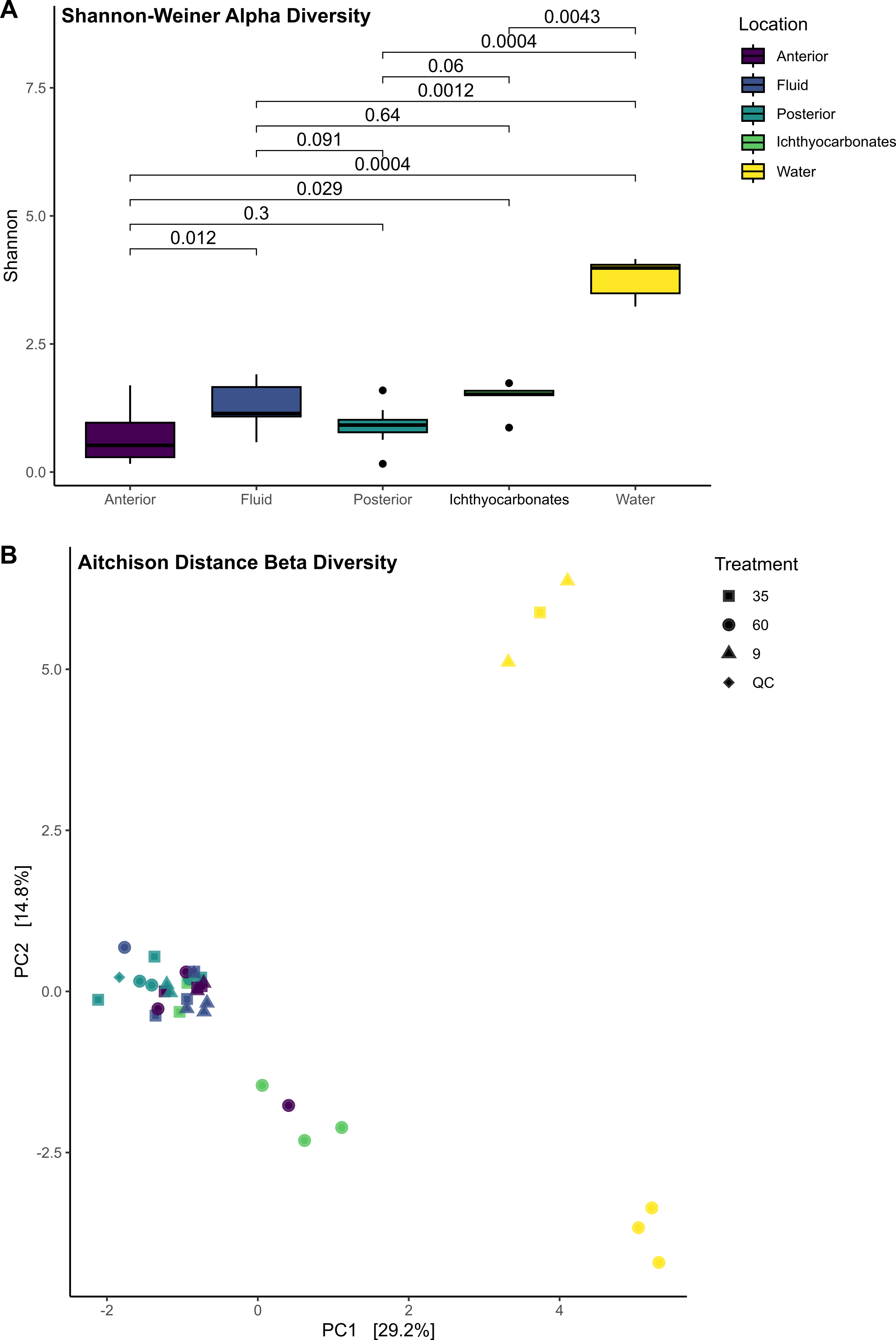

Supplement: S1 Fig — (B) Aitchison Distance beta diversity PCA for samples collected in this study. Shapes represent salinity treatment. Colors represent sample region (location within toadfish gut). (PNG) [file pbio.3003764.s001.png]

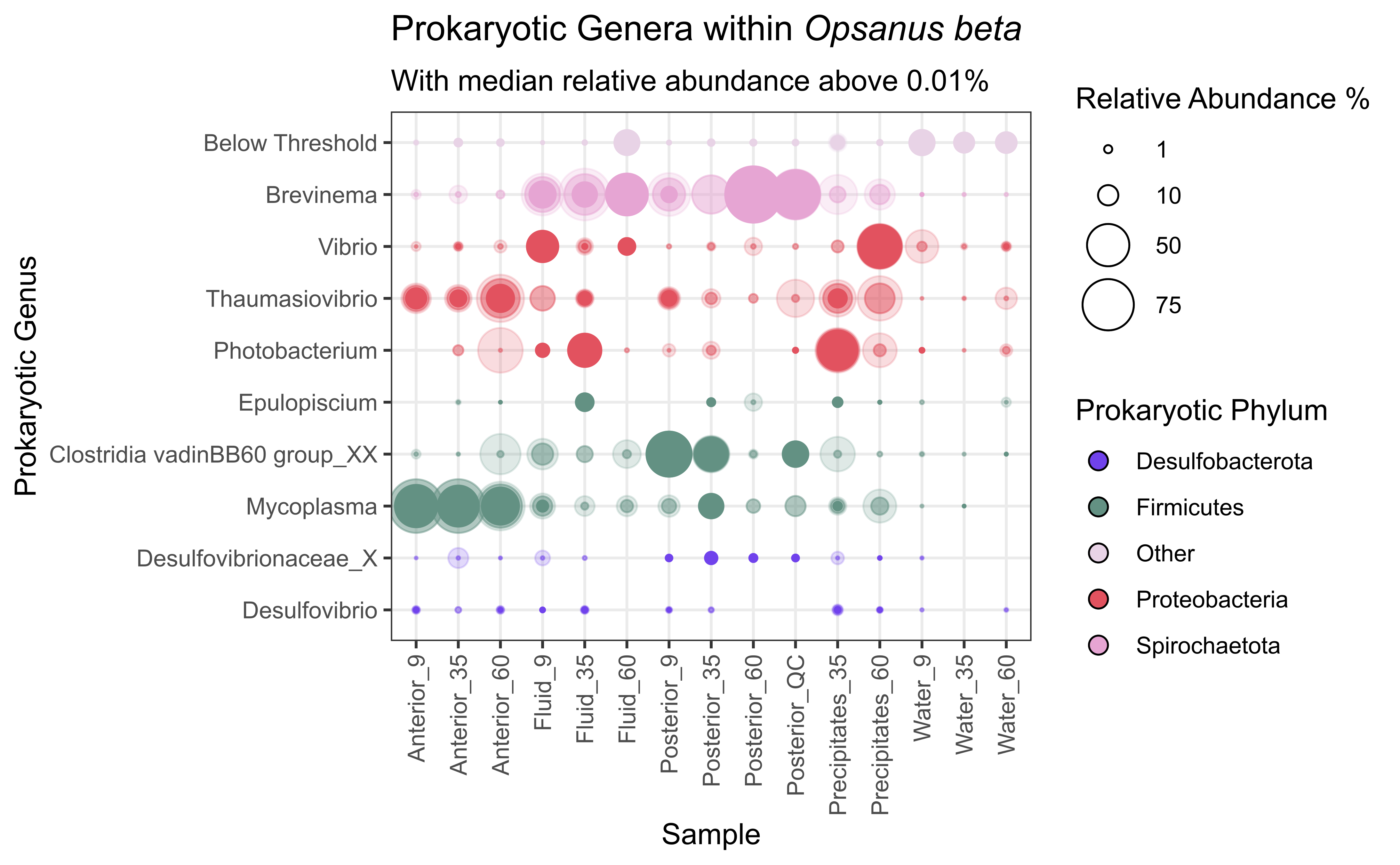

Supplement: S2 Fig — Only genera with a mean relative abundance (MRA) above 0.01% are shown. Genera are colored by phylum, with upper bound and lower standard deviations indicated by bubble shading. IS – incertae sedis. The data underlying this figure can be found on Zenodo (https://doi.org/10.5281/zenodo.18867155). (PNG) [file pbio.3003764.s002.png]

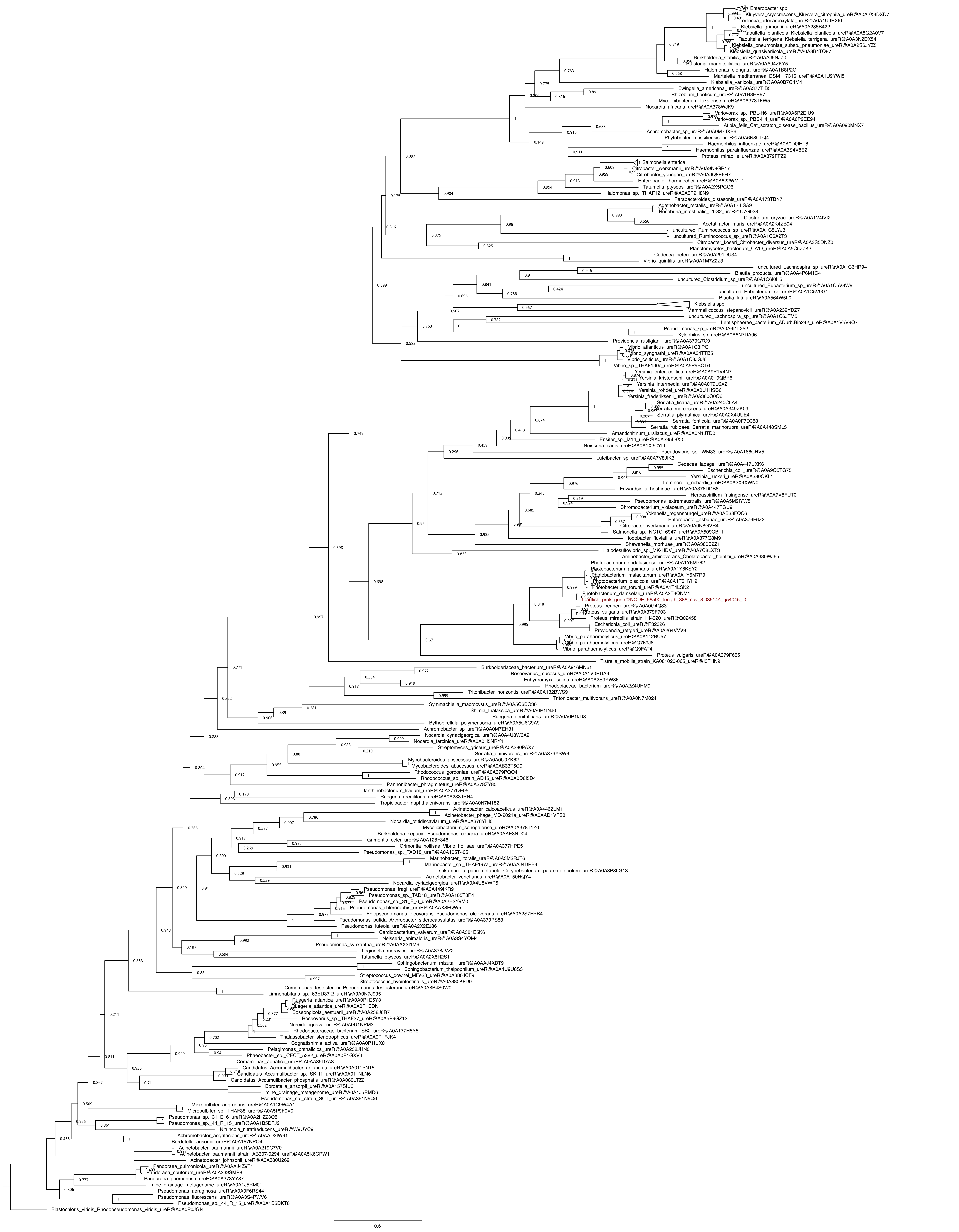

Supplement: S3 Fig — A subset of this tree is used for Fig 3A. The original tree file can be found on Zenodo (https://doi.org/10.5281/zenodo.18867155). (PNG) [file pbio.3003764.s003.png]
